# *In vitro* Validation of Chimeric β-Galactosylceramidase Enzymes With Improved Enzymatic Activity and Increased Secretion

**DOI:** 10.3389/fmolb.2020.00167

**Published:** 2020-07-21

**Authors:** Alessandra Ricca, Federica Cascino, Francesco Morena, Sabata Martino, Angela Gritti

**Affiliations:** ^1^San Raffaele Telethon Institute for Gene Therapy (SR-Tiget), IRCCS San Raffaele Scientific Institute, Milan, Italy; ^2^Vita-Salute San Raffaele University, Milan, Italy; ^3^Department of Chemistry, Biology and Biotechnology, University of Perugia, Perugia, Italy

**Keywords:** globoid cell leukodystrophy, lentiviral vectors, chimeric GALC enzyme, neural stem/progenitor cells, hematopoietic stem/progenitor cells, gene therapy

## Abstract

Globoid Cell Leukodystrophy (GLD) is a lysosomal storage disease (LSD) caused by inherited defects of the β-galactosylceramidase (GALC) gene. The infantile forms display a rapid and aggressive central and peripheral nervous system (CNS and PNS) dysfunction. No treatments are available for GLD patients. Effective gene therapy (GT) strategies for GLD require a safe and widespread delivery of the functional GALC enzyme to all affected tissues/organs, and particularly to the CNS. The use of chimeric lysosomal enzymes with increased secretion and enhanced transport across the blood-brain barrier (BBB) that boost the efficacy of GT approaches in pre-clinical models of similar neurodegenerative LSDs may benefit GLD as well. Here, we tested the safety and biological efficacy of chimeric GALC enzymes engineered to express an alternative signal peptide (iduronate-2-sulfatase – IDSsp) and the low-density lipoprotein receptor (LDLr)-binding domain from the Apolipoprotein E II (ApoE II) in GLD murine neural and hematopoietic stem/progenitor cells and progeny, which are relevant cells types in the context of *in vivo* and *ex vivo* GT platforms. We show that the lentiviral vector-mediated expression of the chimeric GALC enzymes is safe and leads to supranormal enzymatic activity in both neural and hematopoietic cells. The IDSsp.GALC shows enhanced expression and secretion in comparison to the unmodified GALC. The chimeric GALC enzymes produced by LV-transduced cells reduce intracellular galactosylceramide (GalCer) storage and effectively cross-correct GLD murine neurons and glial cells, indicating that the transgenic enzymes are delivered to lysosomes, efficiently secreted, and functional. Of note, the expression of LDLr and LDLr-related proteins in GLD neurons and glial cells supports the exploitation of this system to enhance the GALC supply in affected CNS cells and tissues. These *in vitro* studies support the use of chimeric GALC enzymes to develop novel and more effective GT approaches for GLD.

## Introduction

Globoid cell leukodystrophy (GLD) is a neurodegenerative lysosomal storage disease (LSD) due to the inherited deficiency of β-galactosylceramidase (GALC). The infantile forms (85–90% of all cases) display an unrelenting and aggressive disease progression with a severe central and peripheral neurological deterioration that poses major issues for the development of effective treatments (Wright et al., 2017; Beltran-Quintero et al., 2019).

The blood-brain barrier (BBB) is a complication for enzyme replacement therapies based on the infusion of the recombinant protein. The rationale of applying Hematopoietic Stem Cell Transplant (HSCT) in GLD as well as in other LSDs relies on the ability of transplanted cells to infiltrate the affected central and peripheral nervous system (CNS, PNS) and organs providing functional enzyme, reducing inflammation, and counteracting tissue damage. Indeed, HSCT may improve the clinical outcomes of GLD affected children if performed soon after birth. However, it fails to cure severe motor and cognitive damage (Escolar et al., 2005; Duffner et al., 2009; Wasserstein et al., 2016). GLD is currently considered an untreatable disorder.

In this scenario, gene therapy (GT) represents the most promising approach to address GLD pathology. *In vivo* GT strategies employing adeno-associated vectors (AAV) and lentiviral vectors (LV) to deliver a functional GALC enzyme benefit GLD mice (Rafi et al., 2012; Lattanzi et al., 2014), dogs (Bradbury et al., 2018) and non-human primates (Meneghini et al., 2016). These proof-of-concept studies support the human application of *in vivo* GT. Still, the clinical translation of this approach has to face major safety and efficacy issues related to the pervasive distribution of the vector and/or the transgene that is needed to supply high levels of the corrective enzyme in the human CNS and periphery (Bongarzone et al., 2016; Ricca and Gritti, 2016).

*Ex vivo* LV-mediated GT with autologous hematopoietic stem/progenitor cells (HSPCs) engineered to express the therapeutic gene product (HSC-GT) provides a therapeutic benefit in LSD mouse models (Biffi et al., 2006; Langford-Smith et al., 2012; Visigalli et al., 2016; Ellison et al., 2019) and ameliorates the clinical conditions of patients in several genetic diseases (Cartier et al., 2009; Aiuti et al., 2013; Ferrua et al., 2019; Marktel et al., 2019) including metachromatic leukodystrophy (MLD) (Biffi et al., 2013; Sessa et al., 2016), which shares with GLD the early onset and severe neurological involvement. The moderate benefit of HSC-GT in GLD mice might depend on the poor GALC overexpression achieved in HSPCs and progeny, possibly coupled to modest enzyme secretion and/or insufficient uptake by GLD cells (cross-correction), particularly in the CNS (Gentner et al., 2010; Ungari et al., 2015; Weinstock et al., 2020). Combined cell/GT strategies designed to address the complex multi-organ GLD pathology significantly increase the lifespan of GLD models (Hawkins-Salsbury et al., 2015; Rafi et al., 2015b; Ricca et al., 2015). Still, the combined treatments tested so far fail to provide effective enzymatic reconstitution and full correction of pathology in CNS tissues (Mikulka and Sands, 2016; Ricca and Gritti, 2016). Thus, enhancing the therapeutic potential of GALC together with developing platforms for improved CNS targeting are crucial areas of intervention to improve treatment strategies for GLD.

Modification of the signal peptide (sp) to increase protein biosynthesis/secretion (Owji et al., 2018) and/or addition of different receptor binding sites to favor the transport across the BBB may increase the bioavailability of lysosomal enzymes and boost the efficacy of GT approaches to treat neuronopathic LSDs (Sorrentino et al., 2013; Wang et al., 2013; Böckenhoff et al., 2014; Gleitz et al., 2018). Thus, the use of chimeric GALC enzymes may be key to successfully translate GT approaches to GLD patients. Still, the few studies describing the development of modified GALC enzymes in basic and pre-clinical studies in GLD mice lack a comprehensive assessment of safety and efficacy in therapeutically relevant cell types, i.e., HSPCs and neural cells (Zhang et al., 2008; Hu et al., 2016; Pan et al., 2019).

Here, we report the generation of novel LVs encoding constructs in which the murine GALC gene is engineered by (i) replacing the GALCsp with that of the highly secreted Iduronate 2-sulfatase (IDSsp), to enhance GALC secretion; (ii) adding the Apolipoprotein E II (ApoE II) binding domain for the low-density lipoprotein receptor (LDLr), to favor enzyme transport across the BBB. We test the safety and biological efficacy of these chimeric GALC enzymes in comparison to their unmodified counterpart in HSPCs and neural stem/progenitor cells (NPCs) isolated from Twitcher (TWI) mice (the most common GLD murine model), which represent the effector and target cells, respectively, in GT approaches. Besides, we evaluate the expression and modulation of LDLr and LDLr-related proteins in NPCs and progeny, in the perspective of their exploitation to enhance the supply of chimeric GALC enzymes in CNS tissues.

## Materials and Methods

### Lentiviral Vectors

The plasmids codifying for murine GALC.mCherry (GALC-CH), IDS.GALC-CH and IDS.GALC-CH.APO were synthesized by Gene Script (New Jersey, United States). mCherry sequence was inserted downstream of the murine GALC cDNA using the following linker: ACGCGTACGCGGCCGCTCGAG (TRTRPLE). Transgene expression was driven by the human phosphoglycerate kinase (hPGK) promoter. VSV-pseudotyped third-generation LVs were produced by transient four-plasmid co-transfection into 293T cells and purified by ultracentrifugation, as described (Amendola et al., 2005; Vigna et al., 2005). Expression titers and infectivity of vectors were assessed as previously described (Ornaghi et al., 2020).

Titer (TU/ml) and infectivity (TU/ng) for the different LV batches are the following:

GALC-CH: 5.08 × 10^9^ TU/ml; 4.57 × 10^4^ TU/ng

IDS.GALC-CH: 2.55 × 10^10^ TU/ml; 2.3 × 10^5^ TU/ng

IDS.GALC-CH.APO: 5 × 10^9^ TU/ml; 1.2 × 10^5^ TU/ng

### Establishment of Murine Neural Stem/Progenitor Cell (NPC) Lines

Murine NPC lines were established from the subventricular zone (SVZ) of the forebrain lateral ventricles of neonatal wild type (WT) and Twitcher (TWI) mice (C57BL/6 background; tissues from 2–3 mice were pooled to generate one line) as previously described (Gritti et al., 2009). Primary cells were plated in DMEM/F12 (1:1) medium supplemented with EGF2 and FGF2 (20 ng/ml, Peprotech; growth medium) to generate neurospheres that were collected and serially passaged using the same culture conditions to establish NPC lines. We established independent cell lines from TWI and WT mice (*n* = 2–3 lines/group) and used serially passaged NPC lines (passage 4–15) in all the experiments.

### Lentiviral Vector-Mediated Gene Transfer in NPCs

We transduced WT and TWI NPCs using optimized protocols (Ricca et al., 2015). Briefly, serially subcultured neurospheres were dissociated to single cells and plated in growth medium (proliferating conditions) (30,000 cells/cm^2^). Eight hours (h) after plating, cells were incubated overnight (o/n) with LVs at Multiplicity of Infection (MOI) of 25, 50, and 100. LV-transduced cells were cultured for an additional 4–6 days in fresh medium to obtain neurospheres that were further subcultured to establish stable LV-transduced NPC lines. LV-transduced NPCs used for the experiments were analyzed for proliferation and multipotency.

### Differentiation of NPCs Into Neurons/Glia

Serially passaged neurospheres were dissociated and single cells plated (40,000 cells/cm^2^) onto Matrigel (SACCO) in growth medium. After 2 days we collected proliferating NPCs for molecular and biochemical analysis or exposed them to FGF2-containing medium (48 h) and to a mitogen-free medium added with 2% fetal bovine serum (FBS, Euroclone) for 5 days, to promote neuronal and glial differentiation. The cell-type composition in differentiated cell cultures was assessed by immunofluorescence analysis using lineage-specific markers (Gritti et al., 2009). Primary and secondary antibodies used are listed in Supplementary Table 1.

### Isolation and LV-Mediated Gene Transfer in Hematopoietic Stem/Progenitor Cells (HSPCs)

Lineage negative HSPCs were isolated from TWI and WT adult mice (4–8 weeks) and plated (100,000 cells/cm^2^) in StemSpan serum-free medium (Stemcell) supplemented with cytokines in the presence of LVs, as previously described (Gentner et al., 2010). After LV transduction (MOI 50 and 100 for 12 h), HSPCs were washed, counted and plated for the CFC assay (4,000 cells/ml in Methocult – StemCell) or to obtain liquid cultures (LC), as previously described (Gentner et al., 2010; Ornaghi et al., 2020). After 14 days, we counted the number of colonies (CFC assay) and evaluated the VCN and enzymatic activity in LC.

### Quantification of Vector Copy Number (VCN)

Genomic DNA (gDNA) from murine NPCs and HSPCs was extracted from cellular pellets (mini kit Qiagen), following the manufacturer’s instructions. gDNA was quantified by 260/280 nm optical density (OD) reading on the NanoDrop ND-1000 Spectrophotometer (Euroclone). The VCN was quantified by quantitative droplet dd-PCR, as described (Ornaghi et al., 2020).

### Total mRNA Extraction and Real-Time RT-PCR

Brain tissues were treated with Qiazol and homogenized. Total RNA was extracted using the RNeasy Lipid Tissue mini Kit (Qiagen). Total RNA from cellular pellets was extracted by RNeasy mini Kit (Qiagen). RNA was quantified using NanoDrop ND-1000 Spectrophotometer. Reverse transcription was carried out using 1 μg of total RNA and the QuantiTect Reverse Transcription Kit (Qiagen) following the provider’s instructions.

qPCR was performed in Optical 96-well Fast Thermal Cycling Plates(Applied Biosystems) on ViiA7 Real-Time PCR System (Applied Biosystems), using the following thermal cycling conditions: one cycle at 95°C for 15 min, 40 cycles at 95°C for 50 s and 60°C for 1 min. Each sample was run in duplicate in a total volume of 12.5 μl/reaction, containing 6.25 μl 2× Universal PCR Master Mix (Applied Biosystems), 1 μl of template cDNA and 1.25 μl of probe and primers (TaqMan Gene Expression Assays, Applied Biosystems) listed below. Raw data were extracted using the SDS 2.2.1 software (Applied Biosystems) and the relative expression of mRNA for the target genes was calculated by the comparative CT (ΔΔCT) method, using gapdh as housekeeping gene (Applied Biosystems). The fold change was expressed as 2^–ΔΔCT^.

Probe and primers (Thermo Fisher scientific):

M6Pr Assay ID: Mm04208409_gH

LDLr Assay ID: Mm01177349_m1

Lrp1 Assay ID: Mm00464608_m1

Lrp2 Assay ID: Mm01328171_m1

Gapdh Assay ID: Mm99999915_g1

### Cross-Correction of TWI NPC-Derived Neuronal/Glial Progeny

The supernatant of (i) LV-transduced TWI NPC-derived neurons/glial cells and (ii) untransduced (UT) WT and LV-transduced TWI HSPCs (liquid culture) (*donor cells*) was collected and used to treat TWI NPC-derived neurons/glial cells (*acceptor cells*) for the last 72 h of culture. UT TWI cultures were treated with fresh medium as control. Donor and acceptor cells were collected for analysis (immunofluorescence and GALC activity).

### GALC Activity Assay

GALC activity in murine neural cells (NPCs and differentiated cells) and HSPCs was measured as previously described (Martino et al., 2009). We analyzed *n* ≥ 2 samples in *n* = 1–3 independent experiments. UT WT and TWI cells were included in all the experiments as controls.

### Treatment of NPCs With Statins

WT NPCs were plated (60,000 cells/cm^2^) in growth medium for 24 h. Cells were then exposed to Simvastatin (Sigma-Aldrich, S6196-25 mg) or Pravastatin (Sigma-Aldrich, P4498-25 mg) at 1 and 5 nM for 24 h. Cells were collected for analysis (western blotting and Real-time RT-PCR). For the membrane-bound LDLr protein analysis, 5 × 10^6^ TWI NPCs were plated in growth medium in T75 for 24 h and treated with Simvastatin 1 nM for 24 h. Cells were collected for Cell Surface Protein Biotinylation and Avidin Pull-Down and western blotting analysis.

### Immunofluorescence (IF) Analysis

Immunofluorescence analysis of cultured neural cells and brain tissues was performed as previously described (Meneghini et al., 2017; Ornaghi et al., 2020). Primary and secondary antibodies used are listed in Supplementary Table 1.

Samples were visualized with a Nikon Eclipse E600 microscope and images were acquired at 20× or 40× magnification (scale bars are specified in the legends to figures) with Nikon DS Ri-2 camera, using NIS-Elements F imaging software (Nikon, Japan). Immunoreactive cells were counted using ImageJ and normalized on the total number of nuclei. We counted cells in 3–5 non-overlapping fields/coverslip (100–200 cells/sample), 2–3 coverslips/experiment, *n* = 1–3 experiments. Confocal images were acquired at 63× magnification with zoom (∼2×) with a Leica TCS SP8 confocal microscope and analyzed with LasX software.

### ImageStream Analysis

LV-transduced HSPCs (LC; 3 × 10^6^ cells) were centrifuged and resuspended in 500 μl of PBS/4% PFA (10 min at room temperature – RT), rinsed in PBS, and incubated with PBS/0.1% Triton X-100 (10 min at RT, mild shaking). After washing in PBS, cells were incubated in blocking solution containing MACS buffer (PBS/0.5% bovine serum albumin, BSA/2 mM EDTA) and 10% NGS (1 h, mild shaking). Cells were incubated o/n at 4°C with primary antibodies (anti mCherry, anti-LAMP1) diluted in blocking solution. Antibody staining was revealed using species-specific fluorophore-conjugated secondary antibodies diluted in MACS buffer/1% NGS (1 h at RT). Nuclei were counterstained with Hoechst (4 μM in PBS). After washing in EDTA 2 mM, pellets were resuspended in 50 μl of MACS buffer. Primary and secondary antibodies used are listed in Supplementary Table 1.

Labeled cells were analyzed using the ImageStreamX MarkII System (Amnis, Luminex). At least 20,000 events were collected for each sample (60× magnification, scale bars are specified in the legends to figures). Images were analyzed using IDEAS 6.2 software. Single-stained samples were acquired with identical laser settings of the samples and used for compensation. Cells in focus were gated using the gradient root mean square feature (Gradient RMS) and single cells were identified using area and aspect ratio features on the brightfield image. Cells with high contrast in the brightfield channel (likely debris or apoptotic cells) were excluded from the analyses. Cells with saturation count > 0 were also excluded. A mask for LAMP1 was created: a first spot mask was used for the identification of spots with a size ≥ 5 pixels and a spot to cell-background ratio ≥ 6. Threshold function of 70% was then applied on the spot mask and a further intensity function was used to exclude all spots ≤ 100. Only cells with intense mCherry signal (an indication of efficient LV transduction) were considered for the analysis. The mask for mCherry was created starting from a Spot mask for the identification of spots with a size ≥ 5 pixels and a spot to cell-background ratio ≥ 6. Threshold function of 70% was then applied and a further intensity function was used to exclude all spots ≤ 150. LAMP1 and mCherry masks were combined to create an overlapping mask and the presence of 0 or at least 1 overlapping mask (proximity or co-localization mask) was quantified in each sample. We analyzed ∼1,000/sample cells on average (28–2,604 cells).

### Live Imaging

WT NPCs transduced with LV.GALC-CH were plated (300,000 cells/cm^2^) onto Matrigel-coated Glass Coverslips (Zeta) and cultured for 48 h in growth medium. Coverslips were transferred in an Oko-Lab stage incubator, visualized at 63× magnification with a Leica TCS SP8 confocal microscope, and analyzed with LasX software.

### Western Blotting (WB)

Cell pellets and tissues were resuspended in 50–200 μl (cells) or 500 μl (tissues) of RIPA lysis buffer supplemented with protease (25×, cOmplete Tablets Roche) and phosphatase (10×, PhosSTOP Roche) inhibitors. Tissues were lysed with a homogenizer. The samples were incubated in ice (15 min), vortexed (few seconds), incubated in ice (15 min), and centrifuged at 4°C 12,000 rcf (15 min). The protein extract was collected from the supernatant. Protein concentration was measured using the DCTM Protein Assay and the MultiskanTM Go Microplate Spectrophotometer. Five to fifteen microgram (μg) of protein were fractionated by SDS-PAGE using NuPAGE^TM^ 4–12% BisTris Protein Gels (Invitrogen) and transferred to nitrocellulose membranes using the iBlot2 Gel Transfer Device (Invitrogen). WB was performed as previously described (Martino et al., 1995). Primary and secondary antibodies are listed in Supplementary Table 1. The membranes were developed using ClarityTM ECL Western Blotting Substrate (Bio-Rad) and the Alliance Western Blot Imaging System (UVItec Limited). Quantification of WB was performed using the ImageJ software as described in section 30.13 of the ImageJ User Guide version 1.43.

Proteins were collected from the supernatant of cultures plated at the same cell density by adding four volumes of acetone and incubating the samples o/n at −20°C. The samples were centrifuged at 4°C, 4,000 rcf (20 min), acetone was removed, and the resulting pellets were resuspended in 50 μl of PBS. Equal volume from different samples was incubated with SDS-PAGE Loading Buffer (NuPage, 10 ml) for 15 min at RT before use in WB analysis.

### Cell Surface Protein Biotinylation and Avidin Pull-Down

NPCs (5 × 10^6^ cells) were plated in growth medium in T75 flasks. Cells were collected after 24 h and centrifuged twice (800 rcf, 5 min/each) with cold PBS, 0.1 mM CaCl_2_, 1 mM MgCl_2_ (PBS-CM, Sigma). Cells were treated with biotin (Thermo Fisher Scientific) in cold PBS-CM (1 mg/ml, 1:200; 1 h at 4°C, mild shaking in darkness). The reaction was stopped by removing the biotin and washing 3 times with cold PBS-CM/100 mM glycine (Sigma). Cells were rinsed with cold PBS-CM and lysed in RIPA lysis buffer supplemented with 10× phosphatase inhibitors and 25× proteases inhibitors (1 h at 4°C in a rotating wheel). Protein concentration was measured using the DCTM Protein Assay (Bio-Rad) and the MultiskanTM Go Microplate Spectrophotometer (Thermo Fisher Scientific). Thirty μg of total lysate was used in WB as control (*total lysate*). The remaining lysate was incubated with Neutravidin Agarose Resin (Thermo Fisher Scientific; 40 μl/sample) and resuspended in RIPA buffer plus 1/5 of inhibitors (400 μl/sample; 4°C, 2 h in mild shaking). Samples treated with Neutravidin Agarose Resin or biotin were used as controls. Samples were washed 3 times with PBS/0.1% Triton X-100 (5 min/each, mild shaking at 4°C) and centrifuged at 6,000 rcf (5 min/each, 4°C). Biotinylated proteins were released by adding 50 μl of 4× reducing SDS-PAGE Loading Buffer (NuPage, 10 ml), heating at 99°C (10 min), and centrifuging at 12,000 rcf (2 min) to collect the supernatant. Pull-down of biotinylated proteins (*membrane fraction*) was immunoblotted by WB. Primary and secondary antibodies used are listed in Supplementary Table 1.

### Statistics

Data were analyzed with Graph Pad Prism version 8.0 for Macintosh and expressed as the mean ± std. error of the mean (SEM) when *n* ≥ 3. Statistical significance was set at *p* < 0.05. The number of samples and the statistical tests used are indicated in the legends of each figure.

## Results

### Generation of Lentiviral Vectors Driving the Expression of Chimeric GALC Constructs

**FIGURE 1:**
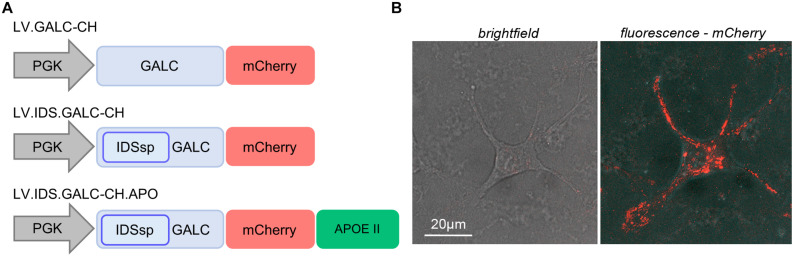
Lentiviral vectors driving the expression of chimeric GALC constructs. **(A)** Schematic of LVs encoding for murine GALC.mCherry (GALC-CH) and GALC-CH-derived chimeric constructs (IDS.GALC-CH and IDS.GALC-CH.APO). **(B)** Representative confocal live imaging picture showing mCherry direct fluorescence in LV.GALC-CH-transduced WT NPCs (brightfield and fluorescent channel). 63× magnification and zoom (∼2×). Scale bar, 20 μm (see also Supplementary Video 1).

We designed and produced third-generation VSV-G-pseudo-typed lentiviral vector (LV) encoding the murine GALC gene fused with the fluorescent reporter mCherry (LV.GALC-CH) under the control of the ubiquitous human phosphoglycerate kinase (PGK) promoter (Figure 1A). The addition of the mCherry fluorescent protein may facilitate GALC protein detection and quantification in western blotting (WB) and immunofluorescence (IF) analysis (see below), as well as in live imaging (Figure 1B and Supplementary Video 1). We have further engineered the GALC-CH construct by (i) replacing the GALC signal peptide (sp) with the sp of the highly secreted IDS (Sorrentino et al., 2013) (LV.IDS.GALC-CH); (ii) adding a flexible linker and a tandem repeat of the human ApoE II receptor-binding region (LV.IDS.GALC-CH.APO) to enhance the blood-brain barrier (BBB) crossing (Wang et al., 2013; Gleitz et al., 2018; Figure 1A; see section “Materials and Methods” for details).

We used these vectors to transduce neural stem/progenitor cells (NPCs) and hematopoietic stem/progenitor cells (HSPCs) derived from wild type (WT) and Twitcher (TWI) mice, which represent relevant target and/or effector cells in GLD pathology and therapy.

### Transduction of NPCs and HSPCs by LVs Expressing Chimeric GALC Is Safe and Results in Supranormal Enzymatic Activity

**FIGURE 2:**
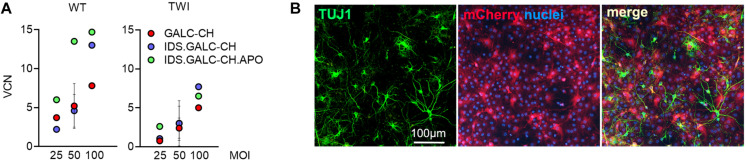
LVs expressing chimeric GALC enzymes efficiently transduce NPCs. **(A)** Vector copy number (VCN) measured in WT and TWI NPCs transduced with the different LVs at 25, 50, and 100 MOI. Data are expressed as the mean (*n* < 3) or the mean ± SEM (*n* ≥ 3). **(B)** Representative IF picture showing neurons (TUJ1, green) expressing mCherry (red) in LV.IDS.GALC-CH.APO-transduced NPC progeny. Nuclei counterstained with Hoechst (blue). 40× magnification. Scale bar, 100 μm.

We transduced WT and TWI NPCs with the different LVs at a multiplicity of infection (MOI) of 25, 50, and 100 using optimized protocols (Ricca et al., 2015). We measured the integrated vector copies/genome (VCN) in LV-transduced NPCs after 4 subculturing passages post-transduction when the non-integrated LV is eliminated. The VCN in LV-transduced NPCs ranged between 0.8 and 14.7 and positively correlated with the MOI used (Figure 2A). LV-transduction was well tolerated by WT and TWI NPCs. All LV-transduced NPC lines (regardless of the VCN) displayed normal proliferation (not shown) and the ability to generate neurons (TUJ1; ∼15%) (Figure 2B), oligodendrocytes (O4; ∼20%; not shown) and astrocytes (GFAP; ∼60%; not shown).

**FIGURE 3:**
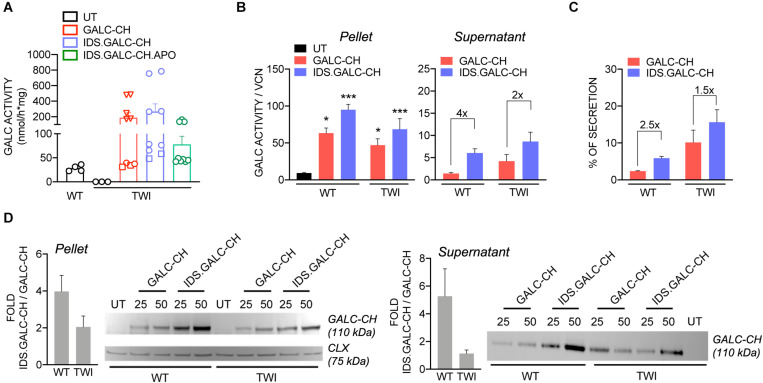
LV-transduced NPCs and progeny express and secrete the chimeric GALC enzymes. **(A)** GALC activity measured in UT and LV-transduced TWI NPCs and UT WT counterparts. Data are the mean ± SEM, *n* = 4 independent experiments in duplicate/triplicate. □ 25 MOI; O 50 MOI; ▽ 100 MOI. **(B)** GALC activity (normalized on VCN) measured in the pellet and supernatant of LV.GALC-CH- and LV.IDS.GALC-CH-transduced WT and TWI NPCs. Data are the mean ± SEM, *n* = 2 independent experiments in duplicate/triplicate. One Way Anova followed by Dunnet’s multiple comparison test, *p<0.05, ***p<0.001 vs. UT WT. **(C)** Percentage of GALC secretion, calculated as GALC activity in the supernatant/total GALC activity (pellet+supernatant) ×100 in LV.GALC-CH- and LV.IDS.GALC-CH-transduced WT and TWI NPCs. Data are the mean ± SEM, *n* = 2 independent experiments in duplicate/triplicate. **(D)** Representative WB and quantification (performed on *n* = 4 blots in different experiments) showing the GALC-CH protein (anti-mCherry antibody) in the pellet and supernatant of WT and TWI NPCs transduced with LV.GALC-CH and LV.IDS.GALC-CH at 25 and 50 MOI. The observed band of 110 kDa indicates the expression of the precursor GALC protein (80 kDa) fused with the mCherry protein (30 kDa). Calnexin (CLX) was used as normalizer (pellet). UT WT and TWI NPCs serve as controls. Data in the graphs are expressed as FOLD: IDS (mCherry/CLX/VCN)/CH (mCherry/CLX/VCN) (pellet) and IDS (mCherry/VCN)/ CH (mCherry/VCN) (supernatant).

The GALC enzymatic activity in LV-transduced TWI NPCs increased at increasing VCN, reaching physiological or supraphysiological (up to 9×) levels, assessed in untransduced (UT) WT NPCs (Figure 3A). These data suggested that the modifications introduced in the GALC sequence did not impact on the enzyme functionality. By normalizing the enzymatic activity on the VCN we showed a consistent advantage of IDS.GALC-CH as compared to GALC-CH in the pellet (∼1.5×) and supernatant of LV-transduced TWI (∼2×) and WT NPCs (∼4×) (Figure 3B). The percentage of secretion – calculated as the enzymatic activity in the supernatant/total enzymatic activity (pellet+supernatant) × 100 – was overall enhanced for IDS.GALC-CH as compared to GALC-CH in both TWI (∼1.5×) and WT NPCs (∼2.5×) (Figure 3C).

WB analysis using an anti-mCherry antibody showed the VCN-dependent increase of GALC precursor protein expression (expected band size 110 kDa: 80 kDa GALC precursor + 30 kDa mCherry) in both the pellet and supernatant of LV-transduced WT and TWI NPCs (Figure 3D). Importantly, the modified GALC enzymes can be detected by an antibody recognizing the endogenous GALC (Lee, 2007; Supplementary Figure 1). This analysis confirmed the trend observed for the GALC activity, showing increased expression of IDS.GALC-CH as compared to GALC-CH protein in the pellet and, to a lesser extent, in the supernatant of WT and TWI NPCs.

**FIGURE 4:**
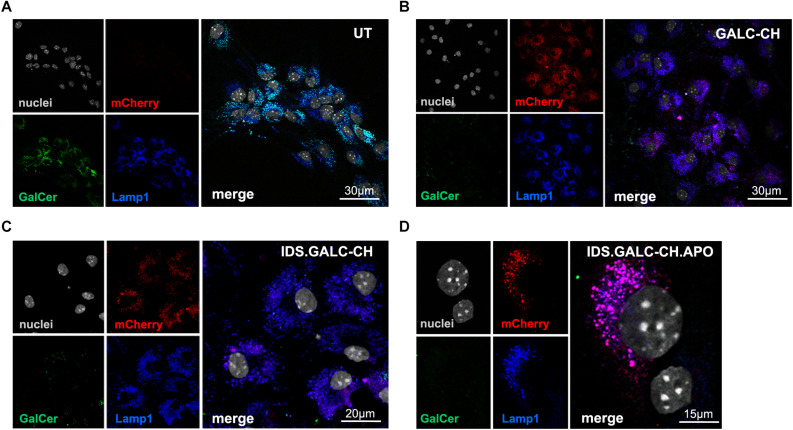
Lysosomal localization of chimeric GALC enzymes. **(A–D)** Representative confocal IF images showing lysosomal localization (LAMP1, blue) of mCherry signal (red) in LV-transduced TWI NPC differentiated progeny expressing GALC-CH **(B)**, IDS.GALC-CH **(C)** and IDS.GALC-CH.APO **(D)**. LV-transduced cells show reduced GalCer storage (green) in comparison to UT TWI controls **(A)**. Nuclei counterstained with Hoechst (gray, pseudocolor); *n* = 1–2 independent experiments, 2 coverslips/group/experiment. 63× magnification and zoom (∼2×). Scale bars: 30 μm **(A,B)**, 20 μm **(C)**, 15 μm **(D)**.

Next, we checked the lysosomal localization of chimeric enzymes and their ability to clear the galactosylceramide (GalCer) storage that accumulates in UT TWI NPC-derived neurons and glial cells (Figure 4A). Confocal IF analysis performed on NPC-derived differentiated cultures showed the co-localization of mCherry^+^ and LAMP1^+^ signal in LV.GALC-CH- (Figure 4B), LV.IDS.GALC-CH- (Figure 4C) and LV.IDS.GALC-CH.APO- transduced cells (Figure 4D), suggesting that the chimeric GALC enzymes are correctly sorted to lysosomes. Importantly, we observed a substantial clearance of GalCer intracellular storage in LV-transduced TWI cells (Figures 4B–D) as compared to UT TWI counterparts (Figure 4A), indicating that the chimeric GALC enzymes are functional.

The safe overexpression and/or secretion of modified GALC enzyme in HSPCs is crucial to favor the enzyme reconstitution of CNS tissues by HSPC-derived myeloid progeny in the context of HSC-GT (Gentner et al., 2010). We isolated HSPCs (Lin- cells) from adult WT (postnatal day 60 – PND60) and TWI mice (PND40, fully symptomatic stage). We transduced WT and TWI HSPCs with the different LVs at MOI 50 and/or 100 using an optimized protocol (Ornaghi et al., 2020). We plated UT and LV-transduced cells for the colony-forming cell (CFC) assay and differentiation in liquid cultures (LCs).

**FIGURE 5:**
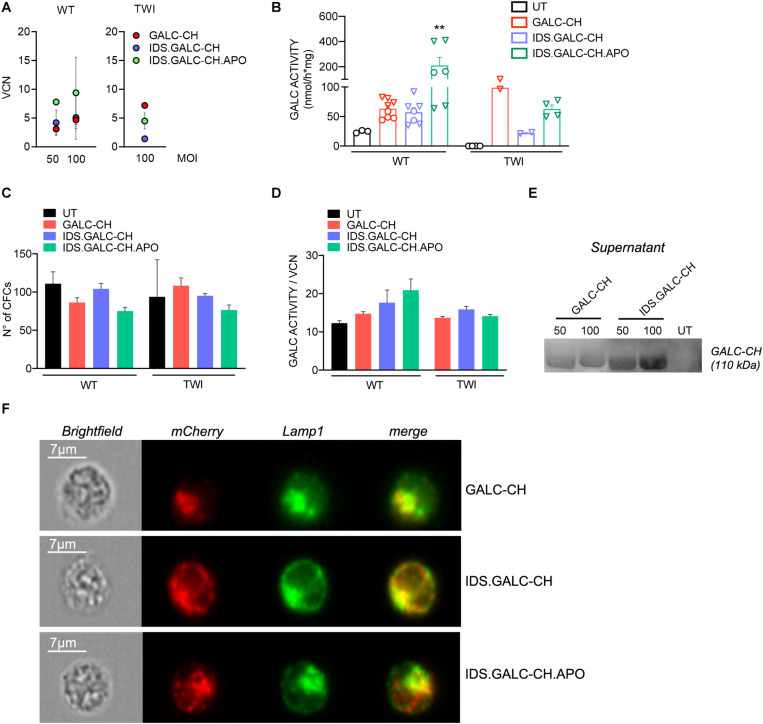
LV-transduced HSPCs and progeny express and secrete the chimeric GALC enzymes. **(A)** Vector copy number (VCN) measured in WT and TWI HSPCs 14 days after transduction with the different LVs at 50 and/or 100 MOI (liquid cultures). Data are expressed as the mean (*n* < 3) or the mean ± SEM (*n* ≥ 3). **(B)** GALC activity measured in UT and LV-transduced WT and TWI HSPCs (liquid cultures). O 50 MOI; ▽ 100 MOI. Data are the mean ± SEM, *n* = 4 independent experiments in duplicate/triplicate. One Way Anova followed by Dunnet’s multiple comparison test, **p<0.01 vs. UT WT. **(C)** Number of colonies (assessed in the CFU assay) generated by UT and LV-transduced WT and TWI HSPCs. Data are expressed as the mean ± SEM, *n* = 4 independent experiments in duplicate. **(D)** GALC activity normalized on VCN measured in UT and LV-transduced WT and TWI HSPCs (liquid cultures). Data are expressed as the mean ± SEM, *n* = 4 independent experiments in duplicate/triplicate. **(E)** Representative WB showing the GALC-CH protein (anti-mCherry antibody) in the supernatant of TWI HSPCs (liquid cultures) transduced with LV.GALC-CH and LV.IDS.GALC-CH at 50 and 100 MOI. The observed band of 110 kDa indicates the expression of the precursor GALC protein (80 kDa) fused with the mCherry protein (30 kDa). Supernatant collected from UT cultures serves as control. **(F)** Representative ImageStream pictures (brightfield and fluorescent channels) showing LAMP1 (green, pseudocolor) and mCherry (red, pseudocolor) proximity (merge) in TWI HSPCs (liquid cultures) transduced with LV.GALC-CH, LV.IDS.GALC-CH, and LV.IDS.GALC-CH.APO. Scale bar, 7 μm.

The VCN in LV-transduced cells ranged between ∼1.4 and 14 (assessed at 14 days, when only integrated LV can be measured; Figure 5A). GALC activity increased at increasing VCN, reaching 2.5× to 4× the normal levels (assessed in UT WT HSPCs) in LV-transduced WT and TWI cells (Figure 5B). This level of GALC-CH, IDS.GALC-CH, and IDS.GALC-CH.APO overexpression was well tolerated by HSPCs, as shown by the comparable number of colonies counted in LV-transduced and UT WT and TWI cells (counted after 14 days; Figure 5C). By normalizing the GALC activity on the VCN we showed a slight advantage of the chimeric GALC enzymes in WT but not in TWI cells (Figure 5D). Of note, WB analysis using an anti-mCherry antibody showed the expression of the GALC precursor protein in the supernatant of LV-transduced HSPCs, indicating that the chimeric proteins were correctly secreted and available in the extracellular environment (Figure 5E).

Next, we assessed the lysosomal localization of chimeric enzymes in HSPCs. We labeled HSPCs with anti-mCherry and anti-LAMP1 antibodies and analyzed labeled cells (and unlabeled controls) taking advantage of ImageStream, an imaging flow cytometer that combines the sensitivity and quantitative power of cytofluorimetric analysis with the qualitative information of the fluorescent microscopy analysis. The results showed a high degree of proximity (an index of co-localization) of the mCherry^+^ and LAMP1^+^ signal in >80% of the LV.GALC- CH-, LV.IDS.GALC- CH-, and LV.IDS.GALC-CH.APO-transduced HSPCs (Figure 5F), suggesting that the chimeric GALC enzymes were correctly sorted to lysosomes.

Overall, these data suggest that NPCs and HSPCs safely overexpress chimeric GALC enzymes, which are correctly localized to lysosomes and secreted in the extracellular milieu. The IDS modification confers a moderate advantage in terms of enzyme expression and secretion in neural cells.

### Chimeric GALC Enzymes Reduce GalCer Storage and Cross-Correct NPC Progeny

The therapeutic efficacy of lysosomal enzymes relies on their capability to be secreted by donor cells and recaptured by deficient cells in the mechanism known as cross-correction (Rastall and Amalfitano, 2017). We evaluated the capacity and efficacy of chimeric GALC enzymes secreted by neural and hematopoietic cells to cross-correct TWI neural progeny, thus modeling the mechanisms acting in the context of CNS-directed GT approaches.

**FIGURE 6:**
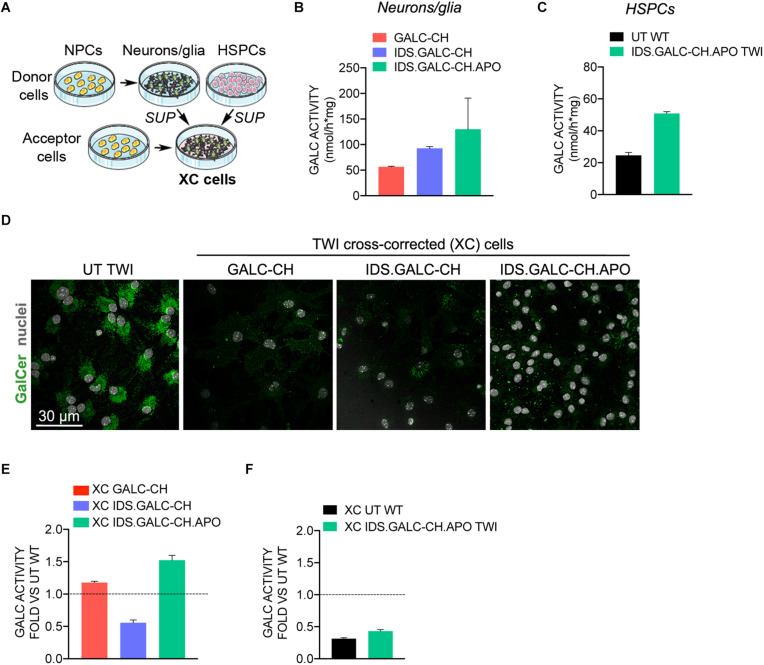
Chimeric GALC enzymes cross-correct TWI NPC-derived progeny. **(A)** Schematic of cross-correction experiments. Donor cells: (i) TWI neuronal/glial cells derived from NPCs transduced with LV.GALC-CH, LV.IDS.GALC-CH, and LV.IDS.GALC-CH.APO; (ii) HSPCs transduced with LV.IDS.GALC-CH.APO (liquid cultures). Acceptor cells: TWI NPC-derived neuronal/glial cells. Acceptor cells are exposed to fresh medium or to the supernatant (SUP) of donor cells during the last 72 h of culture. Donor and cross-corrected (XC cells) are then collected for analysis. **(B,C)** GALC activity in donor cells: TWI neurons/glia transduced with LV.GALC-CH, LV.IDS.GALC-CH, and LV.IDS.GALC-CH.APO **(B)**; WT HSPCs and TWI HSPCs transduced with LV.IDS.GALC-CH.APO (liquid cultures) **(C)**. Data represent the mean with range, *n* = 1 experiment in duplicate. **(D)** Representative confocal IF pictures showing that TWI XC neurons/glia treated with SUP of LV-transduced neuronal/glial cell donors display reduced GalCer storage (green) as compared to UT TWI counterparts. 63× magnification. Scale bar, 30 μm. *n* = 1–2 independent experiments, 2 coverslips/group/experiment. **(E,F)** GALC activity in TWI XC neurons/glia treated with the SUP of neuronal/glial cell donors **(E)** and HSPC donors **(F)**. Data are expressed as fold to WT neuronal/glial cells (dotted line). Data represent the mean with range, *n* = 1 experiment in triplicate.

The schematic of cross-correction experiments is shown in Figure 6A. We treated UT TWI NPC-derived neurons/glial cells (*acceptor cells*) for the last 72 h of culture with the supernatant collected from *donor cells*, namely: (a) TWI NPC derived neurons/glial cells transduced with LVs harboring the chimeric GALC constructs (VCN∼7; 2×–5× the GALC activity measured in WT UT neurons/glia, i.e., 26.0 ± 1.6 nmol/h × mg; *n* = 3) (Figure 6B); (b) UT WT HSPCs or TWI HSPCs transduced with LV.IDS.GALC-CH.APO (VCN∼3; 2× the normal GALC activity measured in UT WT HSPCs) (Figure 6C).

Confocal IF analysis performed in acceptor cells demonstrated the clearance of GalCer storage in cross-corrected (XC) TWI NPC-derived neurons/glia treated with the supernatant of LV-transduced neurons/glia donors (Figure 6D). The GALC activity in XC TWI cultures ranged between 13.27 and 41.53 nmol/h × mg, representing ∼53% (XC IDS.GALC.CH), ∼110% (XC GALC-CH) and ∼150% (XC IDS.GALC-CH.APO) of normal levels assessed in WT neurons/glia (Figure 6E).

The GALC activity measured in XC TWI NPC-derived neurons/glia treated with the supernatant of HSPCs ranged between 7.2 nmol/h × mg (XC WT) and 11.2 nmol/h × mg (XC IDS.GALC-CH.APO TWI), representing ∼20–35% of the normal levels assessed in WT neurons/glia (Figure 6F).

These results suggested that the chimeric enzymes secreted by NPC-derived neurons/glial cells and HSPCs are available in the supernatant for cross-correction and clearance of storage in GALC-deficient neural progeny.

### NPCs and Progeny Express LDLr and LDLr-Related Proteins

Low-density lipoprotein receptor (LDLr) and LDLr-related proteins (LRP1, LRP2) are expressed by brain endothelial cells (Zhao et al., 2016). Thus, the addition of ApoE II binding domain is a promising strategy to favor the BBB crossing of recombinant lysosomal enzymes in LSDs with neurological involvement (Wang et al., 2013; Gleitz et al., 2018). LDLr, LRP1, and LRP2 are also expressed in CNS cells and their role in neurodevelopment and neural cell function is being actively investigated (Marzolo and Farfán, 2011; Auderset et al., 2016; Safina et al., 2016; Engel et al., 2019).

In view of exploiting the LDL-related family of receptors to enhance the uptake of chimeric IDS.GALC-CH.APO in CNS tissues, we investigated the expression of LDLr, LRP1, LRP2, and mannose-6-P-receptor (M6Pr), the canonical receptor mediating the uptake and intracellular targeting of lysosomal enzymes) (Braulke and Bonifacino, 2009) in TWI and WT CNS tissues and NPC-derived neuronal/glial progeny.

**FIGURE 7:**
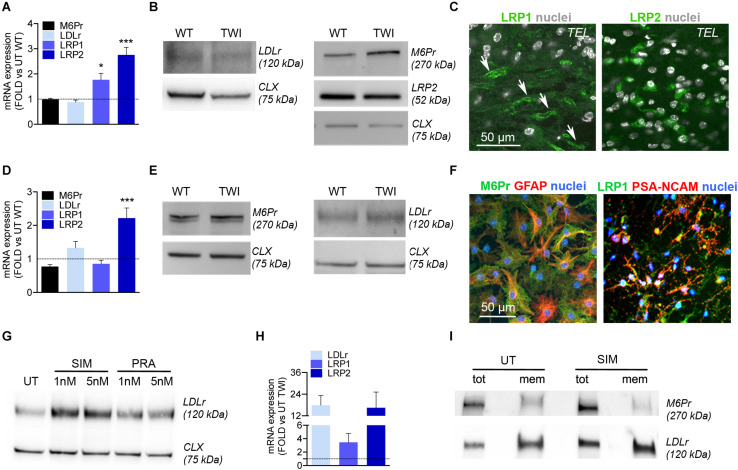
NPCs and progeny express LDLr and LDLr-related proteins. **(A)** Relative mRNA expression of LDLr, LRP1, LRP2, and M6Pr in the telencephalon (TEL) of TWI mice at postnatal day (PND) 40. Data are expressed as fold to WT (dotted line). Data represent the mean ± SEM; *n* = 3 animals/group. ΔΔCT values were analyzed by One Way Anova followed by Dunnet’s multiple comparison test; *p<0.05, ***p<0.001 vs. UT WT. **(B)** Representative WB showing LDLr, LRP2, and M6Pr protein in the TEL of TWI and WT mice at PND 40. Calnexin (CLX) was used as normalizer. **(C)** Representative confocal IF pictures showing LRP1 and LRP2 expression in TEL of symptomatic TWI mice at PND 40. Arrows indicate endothelial cells. Nuclei counterstained with Hoechst (gray, pseudocolor). 40× magnification. Scale bar, 50 μm. **(D)** Relative mRNA expression of LDLr, LRP1, LRP2, and M6Pr in TWI NPC-derived neuronal/glial cell cultures. Data are expressed as fold to WT (dotted line). Data represent the mean ± SEM; *n* = 3 independent experiments. ΔΔCT values were analyzed by One Way Anova followed by Dunnet’s multiple comparison test, ***p< 0.001 vs. UT WT. **(E)** Representative WB showing M6Pr and LDLr protein in TWI and WT NPC-derived neuronal/glial cell cultures. Calnexin (CLX) was used as normalizer. **(F)** Representative confocal IF merged pictures showing M6Pr (green) and LRP1 (green) expression in TWI NPCs-derived astrocytes (GFAP, red) and neuronal progenitors (PSA-NCAM, red). Nuclei counterstained with Hoechst (blue). 40× magnification. Scale bar, 50 μm. **(G)** Representative WB showing LDLr protein in the total lysate of WT NPCs, either UT or treated for 24 h with 5 and 1 nM of Simvastatin (SIM) and Pravastatin (PRA). Calnexin (CLX) was used as normalizer. **(H)** Relative mRNA expression of LDLr, LRP1, and LRP2 in SIM-treated TWI NPCs. Data are expressed as fold to UT TWI (dotted line). Data represent the mean ± SEM; *n* = 2 independent experiments in duplicate. **(I)** Representative WB showing LDLr and M6Pr protein in the total lysate (tot) or membrane (mem) fraction isolated from TWI NPCs, either UT or treated for 24 h with 1 nM of Simvastatin (SIM).

We showed comparable mRNA expression levels of LDLr and M6Pr in adult WT and TWI brain (telencephalon, TEL), and increased expression of LRP1 and LRP2 in TWI as compared to WT brains (Figure 7A). However, WB (Figure 7B) and IF analysis (Figure 7C) showed comparable protein expression of LDLr, LRP1, LRP2, and M6Pr proteins in TWI e WT brain tissues.

To evaluate the expression of these receptors in neurons and glial cells in a system devoid of endothelial cells, we performed Real-time RT-PCR, WB, and IF analysis on TWI and WT NPC-derived differentiated cultures. We showed comparable expression of M6Pr, LDLr, and LRP1 mRNA (Figure 7D) and protein (Figure 7E) in WT and TWI NPC-derived neurons and glia, while LRP2 mRNA expression was significantly increased in TWI as compared to WT cultures (Figure 7D). IF followed by confocal analysis showed that TWI NPC-derived astrocytes (GFAP) and neuronal progenitors (PSA-NCAM) express M6Pr, and LRP1, respectively (Figure 7F).

To test whether the LDLr and LDLr-related proteins expressed by NPC-derived progeny were functional and adaptable, we treated NPC cultures with statins, drugs that are known to enhance the expression of LDLr and LDLr-related proteins. Starting from published protocols (Goto et al., 1997; Pinzõn-Daza et al., 2012) we tested Simvastatin and Pravastatin (1 and 5 nM for 24 h) in pilot experiments using WT NPCs to establish the optimal treatment scheme resulting in the highest upregulation of LDLr, LRP1, and LRP2 expression without adverse toxicity. Simvastatin (1 nM) induced a 2.5-fold increase in LDLr protein expression (Figure 7G) and was chosen to treat TWI NPCs. Simvastatin treatment resulted in a consistent increase of LDLr (18×) and LRP2 (16×) mRNA expression in TWI NPCs (Figure 7H). Also, Simvastatin-treated TWI NPCs showed increased expression (∼2×) of the LDLr protein in the total lysate and, most importantly, in the membrane fraction, without noticeable changes in the expression levels of M6Pr (used as additional control of Simvastatin specificity) (Figure 7I).

Overall these data show that TWI NPCs, neurons and glial cells express functional LDLr and LDLr-related proteins, supporting the idea that this receptor-mediated system may not only favor the transport of IDS.GALC-CH.APO across the BBB but could also mediate GALC uptake in TWI neuronal/glial cells, possibly in addition to the M6Pr-mediated system.

## Discussion

Allogeneic HSCT fails to fully correct the severe CNS pathology of infantile GLD patients, even if performed at the asymptomatic stage (Allewelt et al., 2016), likely because of the insufficient GALC supply provided by HSPC-derived microglia in the brain. Gene therapy (GT) may ensure a stable, robust, and potentially life-long supply of GALC to the CNS and other affected tissues. Still, current GT approaches fail to achieve therapeutic GALC levels and full CNS correction in GLD animal models (Mikulka and Sands, 2016; Ricca and Gritti, 2016). The results of this study address important feasibility, efficacy, and safety issues related to the lentiviral (LV)-mediated (over)expression of functional chimeric GALC enzymes – modified to enhance enzyme secretion and bioavailability – in murine neural and hematopoietic cell types that are the target and/or the effector cells in GT strategies.

The LV-mediated GT platform exploited here provides for high efficiency of gene transfer and low risk of genotoxicity (Biffi et al., 2011; Calabria et al., 2014) and is successfully applied to deliver therapeutic transgenes in pre-clinical studies and in *ex vivo* HSC-GT Phase I/II clinical trials for genetic blood diseases (Aiuti et al., 2013; Ferrua et al., 2019; Marktel et al., 2019) and MLD, a severe neurodegenerative LSD (Biffi et al., 2013). Specifically, the use of gene transfer to boost the production of the Arylsulfatase A (ARSA) enzyme in HSPC-derived myeloid progeny is key to ensure a therapeutic advantage in MLD patients (Biffi et al., 2013; Sessa et al., 2016), in line with the results of pre-clinical studies in MLD mice (Biffi et al., 2006). Still, enzyme overexpression is differently achieved and tolerated, according to the enzyme itself and the target cell type. While ARSA and other lysosomal enzymes (e.g., IDUA) are safely overexpressed to >100× the normal levels without apparent toxicity (Capotondo et al., 2007; Visigalli et al., 2016), only modest GALC overexpression is achieved *in vitro* in HSPCs and progeny (2–3×) (Gentner et al., 2010; Ungari et al., 2015) as well as in neural cells (10–15×) (Lattanzi et al., 2010; Neri et al., 2011; Ricca et al., 2015), suggesting a tight regulation of GALC expression that is only partially elucidated in these cell types. This limitation to GALC overexpression coupled to moderate enzyme secretion by transduced cells and/or insufficient uptake by GLD cells (cross-correction) suggested by previous studies (Ricca et al., 2015; Weinstock et al., 2020) may explain the partial effect of conventional HSCT and the little advantage of HSC-GT in GLD mice (Galbiati et al., 2009; Gentner et al., 2010; Rafi et al., 2015a). Thus, in GLD more than in other LSDs, it is crucial to maximize GALC expression by transduced cells and/or to enhance tissue bioavailability to improve the efficacy of GT approaches.

Studies performed in a murine model of MPS IIIA (a neurodegenerative LSD) have demonstrated the therapeutic efficacy of a chimeric sulphamidase engineered to increase its bioavailability by adding the signal peptide (sp) from the iduronate-2-sulphatase (IDS) and the BBB-binding domain (BD) from the Apolipoprotein B (ApoB) (Sorrentino et al., 2013, 2019). We took advantage of the same approach to generate LVs expressing chimeric GALC enzymes. Specifically, we replaced the GALCsp with the IDSsp and attached the receptor-binding domain of ApoE II. ApoE II shows a higher efficacy as compared to ApoB or ApoE I in enhancing the transport across the BBB of the IDUA enzyme in MPS I mice (Wang et al., 2013) and the enhanced correction of CNS pathology by a chimeric IDS enzyme in a murine model of MPS II (Gleitz et al., 2018). Previous studies reported the generation of chimeric GALC enzymes (Zhang et al., 2008; Hu et al., 2016; Pan et al., 2019). However, their efficacy has been mainly evaluated in cell lines (i.e., 293T, HeLa cells) or fibroblasts (from normal donors and GLD patients), while their safety and efficacy in NPCs, HSPCs, and progeny – therapeutically relevant cells types – has not been extensively studied.

Here, we used optimized transduction protocol resulting in VCN values that are 2–10-fold higher than VCN reported in a previous study evaluating the LV-mediated expression of chimeric GALC enzymes in HSPCs (Hu et al., 2016). Our results show that the addition of the IDSsp and ApoE II peptide did not modify the expression, secretion, or activity of the GALC enzymes *in vitro*. Indeed, the chimeric enzymes produced by transduced NPCs and HSPCs displayed enzyme activity similar or superior to that of the unmodified counterpart. The improvement in LV design and production might explain the improved tolerability and negligible toxicity shown here in LV-transduced murine HSPCs, which are known to be sensitive to GALC overexpression compared to their differentiated myeloid progeny (Gentner et al., 2010). Different strategies exploiting miRNAs (Gentner et al., 2010; Chiriaco et al., 2014) or myeloid-specific promoters (Sergijenko et al., 2013; Ellison et al., 2018) can be applied to control transgene expression in different cell types in the perspective of improving safety and efficacy of HSC-GT approaches for LSDs. The observation that human HSPCs tolerate GALC overexpression better than their murine counterparts (Ungari et al., 2015) supports the clinical development of HSC-GT with improved LVs driving the expression of chimeric GALC enzymes.

The optimal outcome of GT approaches in GLD as well as in other LSDs depends on the ability to boost enzyme bioavailability (production and secretion from donor cells) and cross-correction efficiency (enzyme uptake and delivery to lysosomes of affected cells), particularly in those tissues that are more refractory to correction due to their peculiar anatomy and physiology, such as CNS, PNS, bone, and heart. In line with studies performed in different LSD models (Gleitz et al., 2018; Sorrentino et al., 2019) we modified the GALC enzyme to increase its bioavailability. We show here that the IDSsp modification endows the GALC enzyme with increased secretion as compared to the native counterpart. This advantage is likely determined by the co/post-translational advantage given by the highest affinity of IDSsp to the ribosomal machinery (Staudt et al., 2017). This gain is less clear in HSPCs, further suggesting the cell type-specificity of enzyme production and/or secretion. In contrast, the addition of ApoE II is not expected to help the secretion of lysosomal enzymes. Overall, our results show that both NPCs and HSPCs secrete consistent amounts of the GALC precursor protein (80 kDa), which can be recaptured by deficient GLD neurons/glial cells and, in the lysosome, can be activated by cleavage and assembling of the 50 and 30 kDa forms into the active multimeric enzyme (Hill et al., 2018). Importantly, the use of a GALC-mCherry fusion protein allowed us to provide direct evidence for both secretion and lysosomal localization of the chimeric GALC enzymes in NPCs, HSPCs, and progeny.

The functionality of the GALC-chimeric enzymes produced and secreted by NPCs and HSPCs was ultimately demonstrated by their ability to cross-correct NPC-derived neuronal/glial cells, the specific target in GLD pathology. Cross-corrected neural progeny displayed GALC activity ranging from 25% of normal levels to fully normal levels. Importantly, the full chimeric IDS.GALC.APO enzyme secreted by NPC progeny was as efficient as the other modified enzymes in cross-correcting neural cells, indicating that the lower expression/enzymatic activity – as compared to the other chimeric enzymes – measured in NPCs and progeny was not affecting its function. Our results further confirm that less than physiological enzymatic activity is sufficient to reduce/clear intracellular storage in enzyme-deficient neuronal/glial cells *in vitro*. Whether the therapeutic approaches tested so far in pre-clinical studies or planned for clinical development can ensure these levels of GALC activity in CNS tissues on a “per cell” base remains to be defined.

The uptake of lysosomal enzymes from the extracellular milieu is largely mediated by the mannose-6-P-receptor (M6Pr). While previous studies suggest the contribution of the LDLr and LDLr-related proteins (i.e., LRP1, LRP2) to lysosomal enzyme trafficking (Coutinho et al., 2012; Rothaug et al., 2014; Markmann et al., 2015) few of them addressed the potential involvement of these receptors in the cross-correction of relevant LSD cellular models (i.e., CNS cells). Recent studies suggested the MP6r-independent uptake of ApoB-modified sulphamidase (Sorrentino et al., 2013) and ApoE I-modified GALC (Hu et al., 2016) in patient-derived fibroblasts. In contrast, the ApoE II-modified IDS appears to be recaptured preferentially by M6Pr in endothelial-like cells (Gleitz et al., 2018) suggesting that the mechanism of enzyme uptake might be both transgene- and cell type-specific. The ApoE II modification is expected to favor the GALC uptake by endothelial cells, which express LDLr and related receptors. Importantly, we show here that WT and TWI NPCs and neuronal/glial cells express LDLr and related receptors, and that the expression of these receptors is modulated by statins, suggesting that this system is functional and may be pharmacologically regulated.

The potential advantage provided by the ApoE II sequence in favoring the transport of GALC across the BBB needs to be ultimately studied in GLD mice. We speculate that the fraction of chimeric IDS.GALC.APO secreted by circulating LV-transduced HSPCs could further increase the enzyme supply to CNS tissues. Also, the APO-modified GALC could ensure a more pervasive enzymatic rescue in CNS tissues by the LDLr-mediated direct uptake in neuronal/glial cells. Overall, this functional advantage may reduce the requirement for a high level of GALC overexpression, reducing the vector dose needed to correct HSPCs and thus improving the safety profile of this approach. Also, the expected increased availability of therapeutic GALC levels in CNS tissues soon after HSC-GT may broaden the applicability of this approach to mild symptomatic children. The possibility to use a pharmacological treatment to up-regulate LDLr expression in endothelial cells as well as in neurons/glial cells, as shown here, could be additionally exploited to further enhance GALC availability.

Overall, the results of this study highlight novel tools and complementary mechanisms that can be potentially exploited to promote a more effective reconstitution of GALC activity in CNS tissue. This increased knowledge will facilitate the development of novel GT strategies using modified enzymes to efficiently and timely target different sites of pathology in GLD.

## Data Availability Statement

All datasets/reagents generated for this study are included in the article/Supplementary Material, further inquiries can be directed to the corresponding author.

## Author Contributions

AR and FC contributed to the conception and design of the study, wrote sections of the manuscript, and performed the statistical analysis. AR, FC, and FM performed the experiments. SM supervised the biochemical analysis. AG designed and supervised the study and wrote the manuscript. All authors contributed to the manuscript revision, read and approved the submitted version.

## Conflict of Interest

The authors declare that the research was conducted in the absence of any commercial or financial relationships that could be construed as a potential conflict of interest.
