# Supplementary material for: In vitro Validation of Chimeric β-Galactosylceramidase Enzymes With Improved Enzymatic Activity and Increased Secretion
Source: Front Mol Biosci. 2020 Jul 21;7:167. doi: 10.3389/fmolb.2020.00167 (PMC7396597; doi:10.3389/fmolb.2020.00167)
Supplement: Supplementary file 1 [file Presentation_1.pdf]

Ricca, Cascino et al. "In vitro validation of chimeric β-galactosylceramidase enzymes with improved enzymatic activity and increased secretion"

Supplementary material

Figure S1

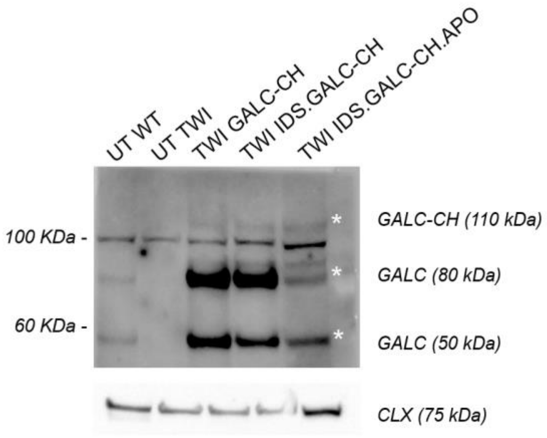

Supplementary figure S1. Representative WB showing GALC protein expression (anti-GALC antibody, CL1021APO, a gift of Dr. WC Lee; Lee, et al. 2007, Lattanzi et al., 2010) in UT WT NPCs, UT TWI NPCs and TWI NPCs transduced with the different LVs (all displaying VCN≈7). The GALC antibody recognizes the GALC-CH protein (GALC precursor 80 KDa + mCherry 30KDa, present only in transduced TWI NPCs), the precursor GALC form (80 KDa), and the mature GALC form (50 KDa). Bands corresponding to the GALC forms are indicated by asterisks. A non-specific band id detected at 100 KDa.Calnexin (CLX) was used as normalizer.

Legend to Supplementary Video S1 (related to Figure 1). Confocal time-lapse images showing mCherry expression in WT NPCs transduced with LV.GALC-CH (100 MOI). NPCs were plated onto Matrigel-coated glass coverslips and cultured for 48 hours in proliferating conditions (growth medium). Coverslips were transferred in an Oko-Lab stage incubator, visualized at 63X magnification with a Leica TCS SP8 confocal microscope, and analysed with LasX software (see materials and methods). Cultures were recorded for 2 min (21 z-stack) in brightfield and direct mCherry fluorescence channel.

Supplementary Table 1. List of primary and secondary antibodies used for immunofluorescence (IF), Wester Blotting (WB), and ImageStream analysis (see materials and methods).

**Primary antibodies**	**Code**	**Dilution**		
		**IF**	**ImageStream**	**WB**
Monoclonal mouse anti-Tuj1	Biolegend, 801202	1:500	-	-
Monoclonal mouse anti-GalCer	Millipore, MAB342	1:300	-	-
Hybridoma rat anti-Lamp1	DSHB, 1D4B	1:300	1:300	-
Polyclonal rabbit anti-LRP1	Santa Cruz, sc16166	1:200	-	-
Polyclonal rabbit anti-M6Pr	Cell Signaling. 14364s	1:300	-	1:300
Polyclonal rabbit anti-mCherry	Abcam, ab167453	1:1,000	1:1,000	-
Polyclonal rabbit anti-LDLr	Biovision, 3839-100	-	-	1:1,000
Polyclonal rabbit anti-LRP2	Santa Cruz, 25470	1:200	-	1:200
Polyclonal rabbit anti-calnexin	Sigma, C4731	-	-	1:3,000
Monoclonal rabbit anti-mCherry	Abcam, ab213511	-	-	1:1,000
Chicken anti-GALC	CL1021APO			1:1,000
**Secondary antibodies**	**Code**	**Dilution**		
		**IF**	**ImageStream**	**WB**
ALEXA 488 anti-mouse	Thermo Fisher Scientific, A11001	1:1,000	1:1,000	-
ALEXA 546 anti-rabbit	Thermo Fisher Scientific, A-11010	1:2,000	-	-
ALEXA 594 anti-rabbit	Thermo Fisher Scientific, A11012	1:2,000	-	-
ALEXA 647 anti-rat	Thermo Fisher Scientific, A21094	1:500	1:500	-
HRP anti mouse	Chemicon, AP124P	-	-	1:5,000
HRP anti rabbit	Chemicon, AP132P	-	-	1:5,000

References

Lattanzi A, Salvagno C, Maderna C, et al. Therapeutic benefit of lentiviral-mediated neonatal intracerebral gene therapy in a mouse model of globoid cell leukodystrophy. *Hum Mol Genet*. 2014;23(12):3250-3268. doi:10.1093/hmg/ddu034

Lee WC, Tsoi YK, Troendle FJ, et al. Single-dose intracerebroventricular administration of galactocerebrosidase improves survival in a mouse model of globoid cell leukodystrophy. *FASEB J*. 2007;21(10):2520-2527. doi:10.1096/fj.06-6169com
